# Analysis of Failure to Finish a Race in a Cohort of Thoroughbred Racehorses in New Zealand

**DOI:** 10.3390/ani6060036

**Published:** 2016-05-25

**Authors:** Jasmine Tanner, Chris Rogers, Charlotte Bolwell, Naomi Cogger, Erica Gee, Wayne Mcllwraith

**Affiliations:** 1 Equine Research Centre, Institute of Veterinary Animal and Biomedical Sciences, Massey University, Palmerston North 4474, New Zealand; jaztanner@gmail.com (J.T.); c.bolwell@massey.ac.nz (C.B.); e.k.gee@massey.ac.nz (E.G.); 2 Epicentre, Institute of Veterinary Animal and Biomedical Sciences, Massey University, Palmerston North 4442, New Zealand; n.cogger@massey.ac.nz; 3 Equine Orthopaedic Research Center, Colorado State University, Fort Collins, CO 80523, USA; Wayne.Mcilwraith@ColoState.EDU

**Keywords:** horse, thoroughbred, horse racing, injury, jockey, falls

## Abstract

**Simple Summary:**

Overall, the failure to finish rate in New Zealand, 2.88 per 1000 horse starts (95% CI 2.64–3.12), was lower than international figures for race day catastrophic injury. Racing and environmental variables such as horse experience, race distance and season were associated with failure to finish a race. Catastrophic injury accounted for approximately half the failure to finish events. Jockey falls were positively associated with less experienced jockeys and horses.

**Abstract:**

The objective was to describe the incidence of failure to finish a race in flat-racing Thoroughbreds in New Zealand as these are summary indicators of falls, injuries and poor performance. Retrospective data on six complete flat racing seasons (n = 188,615 race starts) of all Thoroughbred flat race starts from 1 August 2005 to 31 July 2011 were obtained. The incidence of failure to finish events and binomial exact 95% confidence intervals were calculated per 1000 horse starts. The association between horse-, rider- and race-level variables with the outcomes failure to finish, pulled-up/fell and lost rider were examined with a mixed effects Poisson regression model. A total of 544 horses failed to finish in 188,615 race starts with an overall incidence of 2.88 per 1000 horse starts (95% CI 2.64–3.12). The incidence of failure to finish horses across each race year showed little variability. In the univariable analysis race distance, larger field size, season, and ratings bands showed association with failing to finish a race. The overall failure to finish outcome was associated with season, race distance and ratings bands (horse experience and success ranking criteria). In the multivariable analysis, race distance and ratings bands were associated with horses that pulled-up/fell; season, apprentice allowances and ratings bands were associated with the outcome lost rider. The failure to finish rate was lower than international figures for race day catastrophic injury. Racing and environmental variables were associated with failure to finish a race highlighting the multifactorial nature of race-day events. Further investigation of risk factors for failure to finish is required to better understand the reasons for a low failure to finish rate in Thoroughbred flat races in New Zealand.

## 1. Introduction

In recent years, there has been much attention focused on the quantification of catastrophic and musculoskeletal injury, and risk factors for these in Thoroughbred flat racing [1]. Epidemiological studies have emphasised the multifactorial nature of musculoskeletal events and the complexity of the issues in reducing the risk factors identified [2,3,4,5]. Furthermore, the pattern of training, track surfaces, racing conditions and regulation (e.g., medication use) vary between racing jurisdictions, resulting in different risk factors and rates of musculoskeletal injury worldwide [1,6]. 

Whilst variation between racing jurisdictions may prevent the application of a single global solution, it does provide the opportunity to examine the moderating effects of the racing industry structure on risk factors for, and rates of, injury. To fully interpret these moderating effects it is important to describe the complexity and structure of the racing system within each jurisdiction. Within New Zealand, the production process preceding race training (growth and development leading to yearling preparation) and the training milestones leading up to the first race start have been well described [7,8,9,10], including description of the tissue response leading to first trial start [11,12]. Annually 5562 horses contribute to the 31,488 starters during the season. Most horses enter race training as two-year-olds and will race until the end of their five years old season. During a season flat racing horses start a median of 5 (IQR 2–8) times over race distances of 1400 m (IQR 1200-1670, [12,13]. The temperate climate of New Zealand permits racing year round on approximately 50 different 1800 m turf tracks. Despite the number of different tracks there is consistency in the general dimensions of the tracks and the going of the racing surfaces throughout the season (few with the racing surface classified as “fast” and the majority described as “good” or “dead”) [14]. 

The interaction of the production process with the progression through training [15], and the subsequent influence attainment of these early milestones has on racing success and longevity further emphasize the complexity of the racing system and the need to understand how variables limit progression of the horse through the racing production cycle [16,17]. 

It is important to consider each event reported (race day injury, fracture, catastrophic musculoskeletal injury) as part of an integrated continuum of the interaction of cyclic load (frequency of high speed/gallop strides) and environmental challenge, and not as discrete entities [18,19]. The complexity of the interaction of cyclic load and tissue response is highlighted by the non-linear and dynamic relationships of canter and gallop exercise accumulated during training with fracture risk [20].

Racing and racing injuries could therefore be regarded as part of a complex system. A complex system is a term used to describe how relationships between parts give rise to the collective behaviors of a system and how the system interacts and forms relationships with its environment [21]. An example of the complexity of the racing system was highlighted in a review article [22] with the example given being the apparent greater risk of fatal musculoskeletal injury on turf rather than dirt races in the United States, which may be compounded by the association of longer races on turf compared to dirt [23]. 

Many racing jurisdictions present detailed racing event data from which the horses failing to finish a race can be identified. As part of the rules of racing the Racing Integrity Unit are responsible for producing a stipendiary stewards’ report for each race, detailing any events that occurred during the race. Failure to finish data incorporate a spectrum of events ranging from jockeys “pulling-up” a horse because it was failing to “run on its merits” and suspected injury through to catastrophic injury and jockey falls. As such, failure to finish data provides a holistic system based measure that includes components of racehorse welfare, jockey safety and confidence of the betting public. Thus failure to finish provides an opportunity to screen the performance of a racing jurisdiction for the reliability and consistency of racing. 

There is little published information on the number of horses failing to finish races in New Zealand or possible associations relating to horses not finishing races. Additionally, there appears to be limited analysis of risk factors for failure to finish data in racehorse populations and there is an opportunity to gain greater understanding of the events that occur during a race that prevent horses from finishing. The aim of this study was to describe the incidence of failure to finish a race and investigate risk factors for failure to finish events pulled up and lost rider in flat-racing Thoroughbreds in New Zealand. 

## 2. Materials and Methods

### 2.1. Data

A retrospective cohort study was used to investigate all Thoroughbred flat race starts in the six years from 1 August 2005 to 31 July 2011; data were obtained from New Zealand Thoroughbred Racing (NZTR). Data available included race date, race track, race number, race class, race distance, track condition (or ‘going’), penetrometer reading, horse name, horse age, horse sex, trainer, trainer location (city), finishing position in race, barrier draw (position in the starting gates), carded weight (weight allocated by race handicapper), carried weight (carded weight less any apprentice weight allowance) and domestic rating (analogous to the British horse racing “official rating” system). 

### 2.2. Case Definitions

A horse start occurred whenever a horse entered the starting gate for a race and the gate was released. Any horse involved in an incident that occurred prior to the release of the starting gate and was deemed a late scratching (non-starter) by race day officials was excluded from the study. A horse could contribute several starts and more than one failure to finish event over the study period. Failure to finish occurred whenever a horse started in a race (*i.e.*, the starting gate was released) and failed to cross the finish line. Failure to finish was classified as: pulled up (when the jockey pulled the horse out of the race), fell (when the horse fell during the race), lost rider (when the jockey was dislodged from the horse), brought down (when the horse fell due to collision with another fallen horse), and ran off (when the horse ran off to the outside of the racecourse). 

### 2.3. Statistical Analysis

Data were structured for analysis in Microsoft Excel 2007 and Microsoft Access 2007 (Microsoft Corporation, Redmond, WA, USA) and screened for errors using exploratory data analysis. Continuous data were assessed for normality using the Shapiro-Wilks test. The continuous variables that were categorised into groups included: weight carried (quartiles), race distance (quartiles) and field size (number of starters in the race) (quartiles). New variables were created for race year, season (spring, summer, autumn, winter), field size and whether or not the jockey had an apprentice allowance. Apprentice jockeys in New Zealand claim a weight reduction (allowance) on the handicap weight of horses they ride depending on previous experience. Under current rules an apprentice that has 0–5 career wins claims 4 kg, 6–30 wins claims 3 kg, 31–60 wins claims 2 kg and 61–100 wins claims 1 kg [24]. Ratings were categorised based on the ratings bands recognised by the New Zealand handicapping system. Within the rating system a horses is allocated a numerical rating reflecting its relative performance and its eligibility to compete in differing classes/grades of races. The rating is a dynamic measure of performance that is recalculated within two days of a horse’s most recent race start [24] and is analogous to the rating system used by the British Horse Racing Board [25]. Domestic ratings were categorised into ratings bands as recognised by the New Zealand handicapper. Ratings band 50–54 were maiden (non-race winning) horses, ratings band 55–65 included horses that have won one race and two or more race winners with an extended run of poor form, ratings band 66–75 included most two win horses and three win horses with a recent loss of form and four or more win horses with an extended loss of form. Ratings band 76–85 included most three and four win horses and some open class horses with recent poor form. Ratings band 86–115 were open class (elite) horses [24].

The incidence of failure to finish and corresponding binomial exact 95% confidence intervals were calculated and reported as events per 1000 horse starts for all variables. Failure to finish events were sub-categorised into a pulled-up outcome and a lost rider outcome. Poisson regression was used to estimate incidence rate ratios (IRR) with 95% confidence intervals (95% CI) for the outcomes failure to finish, pulled-up, and lost rider within the univariable analysis. Variables showing association (*p* < 0.2) with the outcomes were analysed in multivariable mixed effects Poisson regression models (for each outcome separately) fitted in a backwards step-wise fashion. A postestimation goodness-of-fit test was performed to test for overdispersion in each multivariable model using the Pearson chi-square and deviance chi-square test, then a random effect for horse was added to each model to adjust for clustering at the horse-level. Biologically plausible interaction terms were assessed in the final models. A Kaplan-Meier curve was used to graphically present the failure to finish by race distance. Statistical significance was set at *p* < 0.05 and analysis conducted in STATA 12 (StataCorp, College Station, TX, USA).

## 3. Results

There were 188,616 race starts for 16,646 individual horses during the study period. The data represented 6072 2-year-old starts, 43,228 3-year-old starts and 139,316 4-year-old and older starts. During the study period the horses contributed a median of 7 (IQR 3–16) race starts. There were 544 failure to finish events providing an overall incidence of 2.88 per 1000 horse starts (95% CI 2.64–3.12). There was little variation in the incidence of failure to finish between racing years. The lowest incidence rate was 2.66 per 1000 starts (95% CI 2.13–3.28) in the 2009/10 racing year and the highest was 3.10 per 1000 starts (95% CI 2.52–3.78) in the 2007/08 racing year. There was no significant effect of horse age associated failure to finish, and no significant difference between ages in the older horse category of 6 years old and older. Of the 544 failure to finish events, 507 (93.2%) horses had single events, 17 (6.2%) horses contributed two events, and one (0.6%) horse had three events. Overall there were 269 (49.4%) pulled-up, 72 (13.2%) fell and 179 (32.9%) lost rider events, other failure to finish events were brought down (*n* = 17) and ran off (*n* = 7).

**Figure 1:**
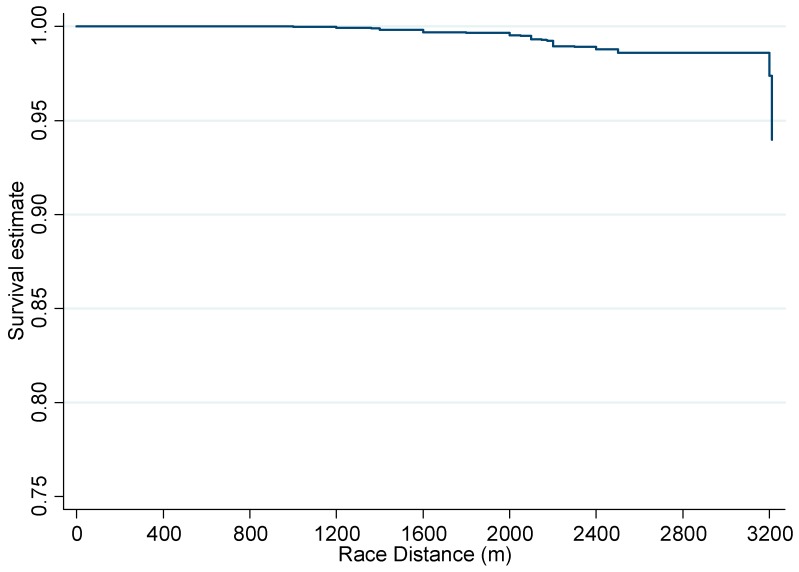
Kaplan-Meier survival curve for incidence of failure to finish and increasing race distance (m) for all Thoroughbred flat race starts in the 2005/06–2010/11 racing years in New Zealand (N = 188,615).

Univariable Poisson regression analysis of the failure to finish, pulled-up and lost rider outcomes are presented in Table 1. Horses racing over a distance of 1671 m or greater were more likely to fail to finish a race or be pulled-up compared to horses racing ≤1200 m (Figure 1). Horses racing in fields of 12–13 runners and 14–18 runners had a higher rate of failure to finish compared to horses racing in fields of 3–9 runners (Table 1). Race year, sex of horse, age of horse, barrier draw and race number on card (order of race at the race meeting) were not significantly associated with failure to finish. 

Multivariable mixed effects Poisson regression models of variables significantly associated with the outcomes failure to finish, pulled-up and lost rider are presented in Table 2. The Pearson goodness of fit statistic for the failure to finish model was *p* = 0.86, for the pulled up model *p* = 0.09, and the lost rider model *p* = 0.60, all indicating good model fit. Season, race distance and ratings band were significantly associated with failure to finish. The failure to finish rate was significantly greater in longer races compared to short races. There was a significant trend for with increasing race distance (*p* < 0.001) with both failure to finish and pulled-up. There was a greater failure to finish rate in autumn compared to spring, and at all rating bands 55 or greater compared to rating band 50–54. The rate of pulled-up was significantly lower in rating bands 66–75 and 86–115 compared to rating band 50–54. 

Season, apprentice allowance and rating band were associated with lost rider in the multivariable model (Table 2). The rate of lost rider events was greater for apprentice allowances compared to no apprentice allowance. Autumn had a significantly lower rate of lost rider events compared to spring, as did rating bands 55–65 and 66–75 compared to rating band 50–54. None of the interaction terms were found to be statistically significant. No variables were found to be significantly associated with the outcome fell within the univariable analysis. 

The addition of the horse as a random effect improved the goodness of fit of the model. The Poisson model was the best fit for the data rather than a negative binomial regression. The large number of horses included as a random effect (*n* = 16,646) prevented the use of Cooks Distance to test for model validity. 

## 4. Discussion

Failure to finish data represent a broad category of events, including musculoskeletal injury, that prevent a horse from completing the race, which does not appear to have been previously reported within flat racing in New Zealand . The failure to finish rates reported in this paper would appear low, and this is reinforced by consideration of international data on race day musculoskeletal injury, which represented a component (~55%) of the New Zealand failure to finish records. Internationally, within the literature, musculoskeletal injury reported on race day ranges from 3.1 per 1000 starts [26] to 4.4 per 1000 starts [27], which is greater than the 2.88 per 1000 starts for failure to finish reported in this paper. As the records utilised in this study were the official racing records it is unlikely that errors in recording or failure to record events has contributed to this low rate. The restricted sampling frame in this study of the racing event may mean that some data outside the racing event, such as the loss of a rider prior to race start, provided some under reporting. It is reported that 47% of jockey falls in New Zealand occur prior to the race start [28]. The data reported in this paper also represents all flat horses racing within New Zealand across a number of years and thus should have minimal bias due to the effect of racing location or seasonal/annual variations in data.

Compared to international studies there is also a low incidence of race day falls by flat race jockeys in New Zealand (2.2/1000) [28] *vs*. 3.7–4.4/1000 for the United Kingdom/ Ireland and France [29] and Australia (4.2/1000) [30], which supports the low failure to finish data reported here. Within the lost rider multivariable model apprentice jockeys claiming a weight allowance were over represented. This observation is in support of data reported out of Australia [31], where the rate of falls by apprentice jockeys was inversely proportional to experience. The dataset did not contain jockey names only the weight a horse was carded and the weight the horse actually carried during the race. This prevented us from including jockey as a random effect within the model, and thus clustering of these events with certain jockeys.

Season (Spring) and race grade (maiden/lower rating horses) were positively associated with lost rider. The race grade effect was also supported by a study of predictors of jockey falls in flat racing in Australia and followed up with the study on early career jockeys [31,32]. The season effect may be due to the horses starting a new campaign in spring and the start of a racing career for young and less experienced horses. Lower grade horses also generally have less racing experience and thus are less predictable and tractable during race riding placing them at greater risk of interference. The lack of interaction between the three variables (rating, season and apprentice jockeys) indicates independent risk and not the presentation of the “perfect storm” of inexperienced jockeys riding “fresh” and “excited” inexperienced horses. 

Contributing factors to the low failure to finish rate may relate to the structure and the type of racing within New Zealand. Racehorses within New Zealand typically run over 1400 m in races with 11 horse fields and have a median of 5 (IQR 2–8) starts per racing season [13]. These parameters *per se*/individually are not uniquely different from other major racing jurisdictions with similar range of field sizes and number race starts reported for flat racing horses in the UK [33] and USA [34]. Distributions for race distances appear sparsely reported within the literature. Most racing jurisdictions appear skewed towards sprint and the lower end of middle distance races, with a mean of approximately 1 mile (1600 m) [23,30,35]. Given the relative international uniformity in many of the racing production parameters reported the low expression of risk identified must relate to some subtle interaction or moderating effect in the production parameters within the complex system. Greater detailed examination of the characteristics of the pattern of racing (changes in racing distance and the timing of the race starts relative to each other) and training (possibly use of the non-totalisator race starts (barrier trials) within the conditioning programme) may help elucidate why the lower than expected rate of risk. 

The apparent low level of race starts, despite a relatively low cost racing structure, may be due to the use of trials (non-totalisator/qualifying races) by racehorse trainers in New Zealand for education and in the final stages of race preparation. A cross-sectional survey of 2-year-old training practices identified the use of trials for education and training milestones within this age group as well as a strong emphasis in 2-year-old training being for education and conditioning purposes rather than with the primary objective of obtaining a race start [10]. 

The presence of the rating band 50–54, which is associated with maiden and 2-year-old racehorses, within the multivariable model indicated that despite these potentially positive characteristics of New Zealand racing there is still an increased rate of failure to finish, and specifically lost rider, associated with younger and more inexperienced horses. This pattern of greater risk with the less experienced/lower grade horses has been reported with jockey falls in Australia [30]. Within the Australian data there was an interaction of jockey experience with horse experience and lower grade races which accentuated the risk, which was not apparent in our dataset. 

Previous studies have shown that racehorse trainers in New Zealand provide horses with voluntary breaks from training (a “spell”) in order to allow the horse time to “strengthen and develop” [10,36]. The willingness of New Zealand trainers and owners to spell a horse may relate to the structure of the racing calendar, which for most horses has a uniform pattern [37]. The consistency of the racing offered throughout the racing season means that provision of a spell may delay a race start but does not impact on the opportunity to have a race start in the appropriate grade/class of race. 

Despite racing on turf tracks within a temperate climate, racetrack surfaces in New Zealand are consistently reported in the good to dead range of going (61% of races, median penetrometer reading 2.3–2.7). It is only in winter that the median going decreases to heavy (penetrometer reading 4.3) [14]. However, even with moderate between season differences in rate of going there was a consistent and limited within season variation in track condition. The consistency in racing surface may be due to focused management at the track level, and at national level the scheduling of the pattern of racing so that the free-draining tracks are used most in winter, which should provide a predictable and relative consistent racing environment for both horse and jockey. The limited variation in racing surface going within a race meeting and across the seasons may explain the lack of significant effect identified for season or going within the multivariable models. The absence of fast tracks (less than 3% of races run in a year) may also be a protective factor in relation to the reported failure to finish rate, as fast tracks have been reported to be associated with an increased risk for race day musculoskeletal injury and fracture [38,39] and race day falls [30]. 

## 5. Conclusions

Overall, the failure to finish rate was lower than international figures for race day catastrophic injury. Racing and environmental variables such as horse experience, race distance and season were associated with failure to finish a race, highlighting the multifactorial nature of race-day events. Investigation of the biological and industry based drivers of the risk factors, particularly season and horse experience are required to identify pragmatic management changes to reduce the risk of failure to finish. Further investigation of risk factors for failure to finish is required to better understand the reasons for a low failure to finish rate in Thoroughbred flat races in New Zealand.

## Figures and Tables

**Table 1:** Univariable Poisson regression for the outcomes failure to finish, pulled-up and lost rider showing incidence rate ratios (IRR) for all Thoroughbred flat race starts in the 2005/06–2010/11 racing years in New Zealand.

				Failure to Finish			Pulled Up				Lost Rider			
Variable	Level	No. of Starts	No. of Failure to Finish Events	Incidence Rate Ratio ^a^	95% CI	*p*-Value ^b^	No. of Pulled up Events	Incidence Rate Ratio ^a^	95% CI	*p*-Value ^b^	No. of Lost Rider Events	Incidence Rate Ratio ^a^	95% CI	*p*-Value ^b^
Race Year	2005/06	29,751	84	Ref		*0.921*	34	Ref		*0.448*	34	Ref		*0.608*
	2006/07	30,574	93	1.08	0.81–1.46	0.599	47	1.37	0.88–2.14	0.165	26	0.75	0.45–1.25	0.262
	2007/08	31,276	97	1.11	0.83–1.49	0.496	42	1.22	0.77–1.93	0.396	35	0.98	0.61–1.57	0.917
	2008/09	33,061	95	1.03	0.76–1.38	0.869	53	1.45	0.94–2.26	0.094	32	0.84	0.52–1.37	0.492
	2009/10	32,349	86	0.95	0.70–1.28	0.730	41	1.15	0.72–1.82	0.557	27	0.73	0.44–1.21	0.218
	2010/11	31,605	89	1.01	0.75–1.36	0.959	52	1.48	0.96–2.3	0.079	25	0.69	0.41–1.17	0.17
Season	Spring	49,620	155	Ref		*0.001*	79	Ref		*0.229*	58	Ref		*0.002*
	Summer	52,647	183	1.12	0.90–1.38	0.320	82	0.98	0.72–1.34	0.917	65	1.06	0.74–1.51	0.742
	Autumn	48,484	106	0.7	0.55–0.90	0.005	55	0.72	0.51–1.01	0.06	31	0.55	0.36–0.85	0.008
	Winter	37,865	100	0.84	0.65–1.08	0.183	53	0.88	0.62–1.25	0.468	25	0.56	0.35–0.9	0.016
Sex	Female	84,011	252	Ref		*0.447*	126	Ref		*0.522*	83	Ref		*0.620*
	Male	104,605	292	0.94	0.79–1.11	0.447	143	0.92	0.72–1.18	0.522	96	0.93	0.68–1.25	0.62
Track Condition	Fast	5,478	19	Ref		*0.134*	11	Ref		*0.462*	5	Ref		*0.820*
	Good	73,231	227	0.9	0.56–1.44	0.653	110	0.75	0.4–1.4	0.369	75	1.13	0.46—2.81	0.787
	Dead	44,481	125	0.81	0.50–1.32	0.398	59	0.66	0.35–1.26	0.208	43	1.07	0.42–2.7	0.889
	Slow	32,310	72	0.64	0.39–1.07	0.088	38	0.59	0.3–1.15	0.12	25	0.85	0.33–2.23	0.747
	Heavy	33,116	101	0.88	0.54–1.44	0.608	51	0.76	0.4–1.47	0.421	31	1.03	0.4–2.65	0.952
Age	2 yo	6,072	19	Ref		*0.936*	8	Ref		*0.098*	7	Ref		*0.420*
	3 yo	43,228	121	0.9	0.55–1.46	0.656	50	0.88	0.42–1.87	0.74	48	0.97	0.44–2.15	0.937
	4 yo	56,374	168	0.96	0.59–1.54	0.853	75	1.03	0.49–2.15	0.94	58	0.9	0.41–1.97	0.787
	5 yo	42,439	116	0.88	0.54–1.44	0.615	63	1.17	0.56–2.47	0.674	34	0.7	0.31–1.58	0.387
	6 yo+	40,503	120	0.96	0.59–1.56	0.864	73	1.45	0.69–3.05	0.324	32	0.68	0.3–1.55	0.361
Apprentice Allowance	No	144,005	398	Ref		*0.079*	213	Ref		*0.279*	116	Ref		*<0.001*
	Yes	44,611	146	1.19	0.98–1.44	0.079	56	0.85	0.63–1.14	0.279	63	1.76	1.3–2.4	<0.001
Race Distance	≤1200 m	49,554	120	Ref		*0.002*	58	Ref		*<0.001*	47	Ref		*0.357*
	1201–1400 m	47,914	129	1.12	0.87–1.43	0.387	47	0.84	0.57–1.24	0.394	55	1.22	0.83–1.81	0.314
	1401–1670 m	44,587	125	1.15	0.89–1.48	0.293	61	1.19	0.83–1.71	0.351	36	0.86	0.55–1.33	0.487
	1671 m+	46,561	172	1.54	1.22–1.95	<0.001	103	1.95	1.4–2.72	<0.001	41	0.93	0.61–1.42	0.739
Weight Carried	46–54.5 kg	55,382	180	Ref		*0.266*	86	Ref		*0.582*	68	Ref		*0.009*
	54.6–55.5 kg	40,677	116	0.87	0.69–1.10	0.261	50	0.78	0.55–1.11	0.17	46	0.92	0.63–1.34	0.678
	55.6–56.9 kg	38,547	103	0.82	0.64–1.05	0.111	54	0.9	0.64–1.26	0.538	29	0.61	0.4–0.95	0.027
	57–76 kg	54,010	145	0.82	0.66–1.03	0.086	79	0.94	0.69–1.28	0.686	36	0.54	0.36–0.82	0.003
Rating Bands	50–54	46,817	176	Ref		*<0.001*	78	Ref		*0.129*	68	Ref		*0.002*
	55–65	45,695	143	0.84	0.67–1.04	0.115	77	1.03	0.75–1.42	0.856	38	0.57	0.38–0.85	0.006
	66–75	57,524	130	0.61	0.48–0.76	<0.001	69	0.73	0.53–1.02	0.066	39	0.47	0.31–0.69	<0.001
	76–85	21,639	58	0.72	0.53–0.97	0.031	27	0.77	0.49–1.2	0.244	19	0.6	0.36–1.01	0.053
	86–115	16,941	37	0.59	0.41–0.85	0.004	18	0.66	0.39–1.12	0.122	15	0.62	0.35–1.09	0.096
Field Size	3–9	43,560	99	Ref		*0.040*	51	Ref		*0.362*	35	Ref		*0.498*
	10–11	42,003	121	1.26	0.97–1.65	0.086	61	1.23	0.85–1.79	0.269	42	1.24	0.79–1.94	0.348
	12–13	45,245	149	1.44	1.12–1.86	0.005	74	1.39	0.97–1.98	0.074	50	1.38	0.89–2.12	0.147
	14–18	57,808	175	1.33	1.04–1.70	0.025	83	1.22	0.86–1.73	0.268	52	1.12	0.73–1.72	0.604
Barrier	1–3	51,029	149	Ref		*0.772*	75	Ref		*0.692*	49	Ref		*0.888*
	4–6	50,625	152	1.03	0.82–1.29	0.806	75	1.01	0.73–1.39	0.957	49	1.01	0.68–1.5	0.956
	7–9	44,443	118	0.91	0.71–1.16	0.441	55	0.84	0.59–1.19	0.332	38	0.89	0.58–1.36	0.596
	10–21	42,519	125	1.01	0.79–1.28	0.960	64	1.02	0.73–1.43	0.904	43	1.06	0.7–1.6	0.784
Race Number	1	16,942	51	Ref		*0.266*	22	Ref		*0.955*	13	Ref		*0.450*
	2	18,389	55	1	0.68–1.46	0.998	25	1.06	0.6–1.89	0.837	22	1.56	0.79–3.1	0.203
	3	18,596	47	0.84	0.57–1.26	0.405	27	1.13	0.64–1.99	0.667	15	1.05	0.5–2.22	0.888
	4	18,840	55	0.98	0.67–1.43	0.897	23	0.95	0.53–1.71	0.87	20	1.38	0.69–2.78	0.364
	5	19,364	51	0.88	0.60–1.30	0.520	33	1.33	0.77–2.28	0.302	13	0.88	0.41–1.9	0.742
	6	19,732	64	1.09	0.75–1.58	0.647	32	1.28	0.74–2.2	0.379	21	1.4	0.7–2.8	0.343
	7	19,925	55	0.93	0.63–1.36	0.691	25	0.98	0.55–1.75	0.956	14	0.92	0.43–1.96	0.833
	8	20,507	59	0.97	0.66–1.41	0.859	30	1.15	0.66–2	0.616	18	1.15	0.56–2.36	0.698
	9	17,487	54	1.04	0.71–1.53	0.844	27	1.22	0.7–2.16	0.482	24	1.8	0.92–3.55	0.088
	10+	18,834	53	0.95	0.64–1.39	0.776	25	1.05	0.59–1.87	0.866	19	1.33	0.65–2.69	0.434

^a^ Adjusted for potential clustering at horse-level; ^b^ Likelihood Ratio Test *p*-value *(italics)* reported as a test, Wald test *p*-value (non-italics) reported as a test for linear trend for continuous variables and ordered categories.

**Table 2:** Results of multivariable mixed effects Poisson regression models of the variables significantly associated with the outcomes: failure to finish, pulled-up, and lost rider (adjusted for potential clustering at horse-level), for all Thoroughbred flat race starts in the 2005/06–2010/11 racing years in New Zealand (N = 188,615).

Variable	Level	Incidence Rate Ratio	95% CI	Wald Test *p*-Value ^a^	LRT *p*-Value ^b^
**Outcome: Failure to Finish**					
Season	Spring	Ref			*0.002*
	Summer	1.08	0.87–1.34	0.502	
	Autumn	0.7	0.55–0.89	0.005	
	Winter	0.82	0.63–1.05	0.117	
Race Distance	≤1200 m	Ref			*<0.001*
	1201–1400 m	1.18	0.92–1.51	0.198	
	1401–1670 m	1.24	0.96–1.60	0.102	
	1671 m+	1.73	1.36–2.20	<0.001	
Rating Bands	50–54	Ref			
	55–65	0.79	0.63–0.99	0.042	*<0.001*
	66–75	0.56	0.44–0.70	<0.001	
	76–85	0.65	0.48–0.88	0.005	
	86–115	0.53	0.37–0.76	0.001	
Horse ^c^					*0.017*
**Outcome: Pulled Up**					
Race Distance	≤1200 m	Ref			*<0.001*
	1201–1400 m	0.88	0.60–1.29	0.517	
	1401–1670 m	1.26	0.87–1.81	0.218	
	1671 m+	2.14	1.53–3.00	<0.001	
Rating Bands	50–54	Ref			*0.019*
	55–65	0.94	0.69–1.31	0.722	
	66–75	0.64	0.46–0.90	0.010	
	76–85	0.65	0.42–1.03	0.064	
	86–115	0.56	0.33–0.95	0.033	
Horse ^d^					*0.009*
**Outcome: Lost Rider**					
Season	Spring	Ref			*<0.001*
	Summer	1.08	0.76–1.54	0.678	
	Autumn	0.56	0.36–0.87	0.009	
	Winter	0.54	0.34–0.87	0.011	
Apprentice Allowance	No	Ref			*<0.001*
	Yes	1.78	1.30–2.43	<0.001	
Rating Bands	50–54	Ref			*0.004*
	55–65	0.59	0.39–0.87	0.009	
	66–75	0.48	0.32–0.71	<0.001	
	76–85	0.61	0.36–1.02	0.058	
	86–115	0.65	0.37–1.16	0.145	
Horse ^e^					*0.005*

^a^ Wald Test *p*-value reported as a test for linear trend for continuous variables and ordered categories; ^b^ LRT: Likelihood Ratio Test; ^c^ = horse level variance 0.54 (0.21–1.34); ^d^ = horse level variance 1.31 (0.59–2.92); ^e^ = horse level variance 1.35 (0.64–2.86).
